# Expert central review in lymphoma diagnosis. Is there a need?

**DOI:** 10.18632/oncotarget.23357

**Published:** 2017-12-17

**Authors:** Camille Laurent, Philippe Gaulard, Pierre Brousset

**Affiliations:** Département de Pathologie, CHU de Toulouse, Institut Universitaire du Cancer de Toulouse, Toulouse, France; INSERM, Centre de Recherche en Cancerologie de Toulouse-Purpan, laboratoire d’excellence TOUCAN, Toulouse, France

**Keywords:** expert review, lymphoma diagnosis, impact on patient care, cost-effectiveness

The effective management of hematopoietic tumours depends on accurate pathological diagnosis, which is mandatory for giving an appropriate treatment for lymphoma patients. Despite the introduction of the WHO classification which offers a uniform scheme for distinguishing the different lymphoma subtypes, the risk of error remains higher than in other areas of pathology, since their diagnosis requires experience and large panel of ancillary tests that are not available in routine laboratories. This difficulty is illustrated by previous studies in (the) UK and USA showing a variable rate of discrepancy on lymphoma diagnosis between expert and non-expert pathologists [1-3]. In 2010, the “Institut National du Cancer” (INCa) established the *Lymphopath* network to provide, prior to treatment, a pathological review by an expert hematopathologist of every newly diagnosed or suspected lymphoma in France. Expert pathologists are selected according to their experience in lymphoma diagnosis – reporting more than 200 lymphoma cases per year – and who are working in one of the 36 academic institutions [4]. The main goal of this network is to render in real-time an accurate lymphoma diagnosis, confirming or revising non expert conclusions for optimal clinical management of patients. Since 2010, the *Lymphopath* network has dealt with more than 70% of all new lymphoma cases in France and provided a prospective expert review for 65 000 samples of newly diagnosed or suspected lymphoma cases. In a recent issue of the Journal of Clinical Oncology, we showed that, over a four-year period, expert review changed the diagnosis in almost 20% of patients with a potential impact on patient care in 17.4% according to the current medical guidelines [4]. Most of the discrepancies were due to misclassifications of lymphoma subtypes resulting in major changes before the clinical management of patients. Previous studies carried out at monocentric or regional levels have reported variable rates of discordance with a potential impact on patient care ranging from 2% to 17% [1-3]. However, it should be noted that in contrast to these studies, *Lymphopath* included a large number of private and non-academic labs (more than 500) on a national scale which may have more limited access to new tools and deal with few lymphoma cases per year, especially of rare and challenging ones (for example T-cell lymphomas).

Another key finding of our study is that cases sent with a formal diagnosis showed a much lower rate of diagnostic changes than cases sent with a provisional diagnosis. This means that when referral pathologists were confident about the diagnosis, the latter was more likely to be accurate. Some discrepancies could result from premature sending of cases by referral pathologists. Indeed, in order to minimize the delays of patients’ management some pathologists prefer to send cases to the expert center with a minimal set of techniques. This could in part explain why we did not observe a significant decline in the overall discordance rate over the four-year period, in contrast to Proctor et al.[2]. But we also believe that training in hematopathology has a long way to go before obtaining significant results. However, encouragingly, we observed that *Lymphopath* promoted the use of more specific antibody clones by referral pathologists as for example anti-cyclin D1/SP4 or anti-BCL2/SP66, thus improving the diagnostic rate of mantle cell lymphoma and follicular lymphoma respectively. Based on our study, we do not think that an expert review should be mandatory in all instances as illustrated by the weakest rate of discordance for cases sent with a formal diagnosis. Further investigations need to determine which level of skill (or training) should be reached and/or which ancillary facilities should be required to allow referral pathologists to select cases to send out for expert review.

Regarding the cost of the *Lymphopath* review, the government’s funding is around 450 000 euros per year. Although this is a significant cost, we have to determine the financial impact of diagnostic changes in therapeutic management. Further economic evaluations are currently ongoing to investigate whether *Lymphopath* increases the cost effectiveness of patient’s care.

Finally, since 2010 *Lymphopath* has provided a large national registry on all lymphoma subtypes in France (to date, over 60 000 lymphomas) which could be useful for large epidemiological studies. For example, *Lymphopath* has shown that angioimmunoblastic lymphoma is the most frequent T-cell lymphomas in France [5]. It also gives the opportunity to identify the frequency of uncommon entities such as plasmablastic lymphoma [6] or the emergence of new provisional entities such as anaplastic large cells lymphoma associated with breast implants [7].

To conclude, our study implies that in around 20% of cases, lymphoma diagnoses are better determined in collaboration with expert pathologists. Based on the upcoming 2016 WHO classification which identifies an increasing number of lymphoma subtypes, it will be even more important to assess accurate diagnoses in the near future especially in the current age of personalized medicine.

## References

[1] Bowen JM (2014). Br J Haematol.

[2] Proctor IE (2011). J Clin Oncol.

[3] LaCasce AS (2008). J Clin Oncol.

[4] Laurent C (2017). J Clin Oncol.

[5] De Leval L (2015). Haematologica.

[6] Laurent C (2016). Haematologica.

[7] Laurent C (2016). Ann Oncol.

